# Environmental co-exposure to microplastics and phenol may influence redox signaling and tumor microenvironment dynamics

**DOI:** 10.7150/jca.135514

**Published:** 2026-08-10

**Authors:** Chung-Yu Lin, Che-Hsin Lee

**Affiliations:** 1 Department of Biological Sciences, National Sun Yat-sen University, Kaohsiung 80424, Taiwan;; 2 Aerosol Science Research Center, National Sun Yat-sen University, Kaohsiung 80424, Taiwan;; 3 Department of Medical Research, China Medical University Hospital, China Medical University, Taichung 40433, Taiwan;; 4 Department of Medical Laboratory Science and Biotechnology, Kaohsiung Medical University, Kaohsiung 80708, Taiwan;

**Keywords:** microplastic, phenol, oxidative stress, mitogen-activated protein kinase signaling, tumor microenvironment.

## Abstract

Environmental exposure patterns have shifted from single-compound exposure to complex co-exposure scenarios. Among widespread pollutants, microplastics (MPs) and phenol may interact biologically despite their distinct physicochemical properties. Phenol is associated with oxidative stress-related toxicity, whereas MPs may serve as potential carriers and redox-active modifiers. This review proposes that MP-induced oxidative stress and phenol-mediated antioxidant disruption may jointly reshape redox-sensitive intracellular signaling under MPs-phenol co-exposure conditions, with mitogen-activated protein kinase (MAPK) pathways proposed as potential convergence nodes. Within this framework, MAPK signaling may link oxidative stress to hypoxia-inducible factor 1α (HIF-1α) stabilization, angiogenic responses, and tumor-associated processes, and may also facilitate NF-κB/STAT3-mediated inflammatory signaling and PD-L1 regulation. Because direct experimental evidence for these integrated mechanisms remains limited, this framework should be regarded as a biologically plausible and testable model rather than a confirmed causal pathway. This review highlights key knowledge gaps and future priorities for evaluating how complex environmental co-exposures may influence cancer progression and immune regulation.

## 1. Introduction

In the context of increasing global environmental pollution, anthropogenic chemical substances and particulate pollutants have become widely distributed in water, soil, air, and food chains, leading to persistent low-level background exposure in both ecosystems and human populations 1, 2. Traditional regulatory frameworks and environmental risk assessments have primarily focused on the acute toxicity and high-dose effects of single pollutants 3-5. However, real-world exposure scenarios are often more complex, typically involving the coexistence and interaction of multiple contaminants, which can alter contaminant transport and bioavailability, thereby challenging conventional single-pollutant risk paradigms 1, 6.

In recent years, the concepts of mixture exposure and carrier-mediated exposure have gained increasing attention, particularly with the rapid accumulation of emerging pollutants such as microplastics (MPs) 7-9. Accordingly, the exposure patterns and biological effects of conventional chemical pollutants require re-evaluation under contemporary environmental conditions shaped by co-existing particulate contaminants. This is particularly important because conventional single-pollutant frameworks may underestimate chemical risks when particulate contaminants potentially modify local exposure dynamics and biological responses.

Therefore, re-examining the biological effects of conventional pollutants under modern co-exposure conditions may provide new insight into their health impacts and underlying molecular mechanisms, while offering a more realistic framework for environmental risk assessment.

## 2. Phenol: a classical yet underappreciated environmental pollutant

### 2.1 Phenol as an underrecognized environmental pollutant

Phenol is an important commodity chemical widely used in petrochemical production, plastics manufacturing, pesticides, pharmaceuticals, and personal care products 10, 11. It also serves as a key precursor for bisphenol A (BPA) and various phenolic derivatives, supporting its sustained industrial demand 12. Its widespread and sustained industrial use suggests that environmental release and background exposure are likely to persist over time 13, 14. Despite this widespread use, phenol has been less frequently discussed as an individual tumor-related environmental pollutant than its phenolic derivatives 15, 16. As an important parent structure of phenolic compounds, however, phenol warrants independent attention from both environmental health and mechanistic perspectives, rather than being regarded merely as a generic precursor or interchangeable member of a broader phenolic group.

### 2.2 Environmental occurrence and exposure characteristics

From an environmental perspective, phenol can enter water, soil, and other environmental media through industrial emissions and wastewater discharge, thereby contributing to continuous background exposure 17, 18. Its relatively high mobility in aqueous environments may facilitate environmental spread and persistent exposure 19, 20. For this reason, phenol has been listed as a priority pollutant under regulatory frameworks, including those of the United States Environmental Protection Agency (EPA) and the European Union (EU) 21, 22. Importantly, this mobility distinguishes phenol from more hydrophobic phenolic pollutants, suggesting that it may follow distinct exposure trajectories and mixed-exposure risk profiles that remain underrecognized in tumor-related environmental research.

However, previous toxicological studies and risk assessments have largely focused on acute toxicity endpoints, such as LC50, and may not adequately reflect the cellular signaling and microenvironment-level effects associated with long-term, sublethal exposure at environmentally relevant concentrations 23-25. A related concern is the possibility of regrettable substitution 26-28. Even when regulations reduce the use or environmental levels of specific chemicals, structurally similar substitutes may retain comparable or even stronger biological effects, suggesting that the underlying chemical backbone contributes importantly to toxicity profiles 29, 30.

Notably, although phenol has received less attention than several phenolic derivatives in cancer-related toxicology, it possesses distinct physicochemical characteristics, including relatively high-water solubility and environmental mobility, which may influence its exposure patterns and biological interactions. In addition, as the parent structure of numerous phenolic compounds, phenol may provide a useful framework for investigating redox-sensitive responses associated with environmentally relevant phenolic pollutants. These considerations support selecting phenol as the primary focus of the present review and highlight the need for its independent evaluation under realistic co-exposure conditions, rather than direct extrapolation from more extensively studied phenolic derivatives.

### 2.3 Evidence gaps and rationale for focusing on phenol

In addition, several recent epidemiological studies have suggested possible associations between phenol exposure, or related exposure indicators, and the risk of certain cancers or cancer-related outcomes 15, 31. It should be emphasized that these findings are mainly based on correlational evidence and are not sufficient to establish clear causal relationships 16, 32. Accordingly, this review considers whether phenol may influence tumor-related phenotypes through redox-sensitive signaling and tumor microenvironment-related mechanisms. Structurally related phenolic compounds are included only as mechanistic analogs and are not treated as equivalent to phenol in causal interpretation, thereby maintaining phenol as the primary focus of this review.

## 3. Microplastics: an emerging yet complex environmental pollutant

### 3.1 Environmental source and characteristics of microplastics

MPs are generally defined as plastic particles smaller than 5 mm in size. Over the past four decades, their environmental abundance has continuously increased, and they are now recognized as emerging pollutants of global concern 33, 34. Primary microplastics are intentionally manufactured particles used in industrial and consumer products, whereas secondary microplastics are generated through the fragmentation of larger plastic materials in the environment 1, 2, 35-37. In most environmental settings, secondary microplastics are considered a major source, with both terrestrial inputs and marine activities contributing to their overall environmental burden 38, 39.

### 3.2 Aging-dependent roles of MPs as sources and carriers of phenolic compounds

Importantly, MPs are not static or inert particles, but undergo continuous aging through ultraviolet radiation, oxidation, and mechanical abrasion. These processes alter their surface properties and reactivity, often through changes in functional groups and biofilm formation 40, 41. During these transformations, MPs contribute to phenolic-compound exposure through two potential pathways. First, they may act as a potential intrinsic source, as phenolic additives can undergo degradation during aging and release phenolic additives or related degradation products 42. Second, they may function as external carriers, with their surfaces and polymer matrices capable of adsorbing organic pollutants and influencing their transport and bioavailability during exposure 43. These findings suggest that MPs may not only contribute to the exposure risk associated with the particles themselves, but may also modify the environmental behavior and exposure patterns of phenolic compounds in environmentally relevant contexts. This dynamic behavior is particularly relevant to co-exposure assessment, as interactions between MPs and phenolic compounds may shift with environmental aging rather than remain constant across exposure settings.

### 3.3 Relevance of MPs to phenol-related co-exposure scenarios

**Figure 1:**
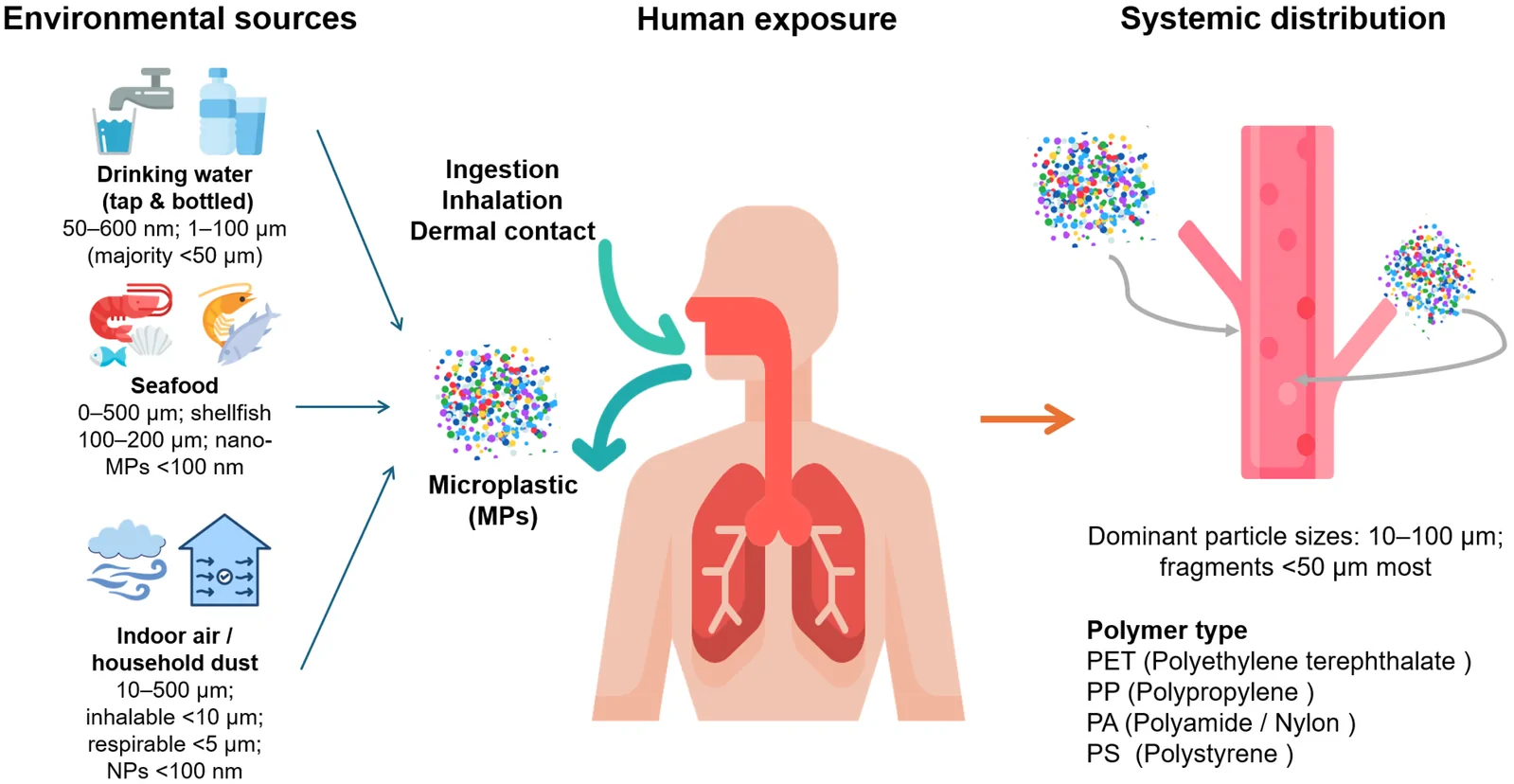
** Real-world microplastic exposure pathways and systemic distribution.** Environmental sources of microplastics (MPs) include drinking water (50-600 nm; 1-100 μm, with the majority <50 μm), seafood (10-500 μm; 100-200 μm; nano-MPs <100 nm), and indoor air or household dust (10-500 μm inhalable; <10 μm respirable; <5 μm alveoli-penetrating particles; nanoparticles <100 nm). These MPs enter the human body primarily through ingestion, inhalation, and dermal contact. Once exposed, MPs of various polymer types (PET, PP, PE, PS, PA) can be absorbed and transported into systemic circulation. Human blood studies report dominant particle sizes of 10-100 μm, with fragments <50 μm being most abundant, indicating that widespread low-level environmental exposure can lead to measurable internal burdens. Arrows illustrate the flow from environmental sources to human exposure routes and subsequent bloodstream distribution.

To summarize environmental characteristics relevant to human exposure, this review outlines the major exposure routes, common size ranges, and dominant polymer types of MPs (Table 1). Correspondingly, Fig. 1 provides a schematic representation of these features, illustrating environmental sources, primary human exposure routes (ingestion, inhalation, and dermal contact), and systemic distribution. In this context, MPs are not discussed as an isolated exposure factor in this review. Instead, they are considered a key framework for understanding phenol exposure under more realistic mixture exposure conditions in modern environments. Collectively, these characteristics suggest that phenol exposure may occur within more complex environmental contexts than those considered in traditional single-pollutant assessments. Consequently, evaluating phenol under co-exposure scenarios involving MPs may provide a more realistic framework for understanding its environmental behavior and potential biological effects.

## 4. Trojan horse effect: a carrier-mediated yet context-dependent exposure mechanism

### 4.1 Physicochemical basis of carrier-mediated transport

The Trojan horse effect is commonly used to describe a carrier-mediated transformation of exposure in studies examining interactions between MPs and environmental contaminants. MPs possess a high surface area and hydrophobic polymer matrices, and their weathering is often accompanied by changes in surface functional groups and biofilm formation 44, 45. These properties enable MPs to adsorb environmental pollutants and transport them into biological systems as particulate carriers, thereby altering their exposure routes and local release dynamics. Pollutants can accumulate on the surface and within the pores of MPs, and upon ingestion or inhalation, may be gradually released in the gastrointestinal tract or intracellular microenvironments, leading to potentially elevated local bioavailable fractions in specific tissues or cellular regions 46, 47. This concept has therefore been widely used to explain how MPs may modify the environmental transport, bioavailability, and exposure characteristics of diverse organic contaminants 48.

### 4.2 Evidence from phenolic analogs

Within this framework, “phenolic compounds” are defined as aromatic compounds containing a hydroxyl (-OH) group, including representative substances such as nonylphenol, bisphenol A (BPA), and butylated hydroxyanisole (BHA) 30, 49. This review focuses primarily on phenol; however, more hydrophobic phenolic compounds are included as mechanistic analogs to compensate for the currently limited direct evidence on phenol under co-exposure conditions. At the physicochemical level, the interaction between phenolic compounds and MPs is governed by mechanisms such as hydrophobic partitioning, π-π interactions, and hydrogen bonding 50, 51. For instance, aromatic structures may interact with plastic matrices through π-π stacking, while phenolic hydroxyl groups can form hydrogen bonds with weathered surface functional groups, influencing adsorption capacity and desorption behavior 52. Collectively, these physicochemical interactions suggest that MPs may alter the environmental fate and exposure patterns of phenolic compounds under specific conditions.

At the biological level, multiple studies have reported enhanced toxicity under co-exposure to MPs and phenolic compounds. For example, co-exposure to nonylphenol and polystyrene microplastics (PS-MPs) in Caco-2 intestinal epithelial cells resulted in greater toxicity than single exposures, consistent with a carrier-mediated exposure effect at the cellular level 53. Similarly, BPA has been shown to exhibit enhanced cytotoxicity and oxidative stress responses under co-exposure with MPs, along with alterations in thyroid function and metabolism in zebrafish models, suggesting that particle-bound phenolic compounds may influence *in vivo* responses 54.

### 4.3 Applicability and limitations for phenol

However, for phenol, which has a relatively simple structure and higher water solubility, the contribution of MPs as a primary transport carrier is likely more limited under typical environmental conditions compared to more hydrophobic phenolic compounds 55. In addition, its carrier-mediated effects are likely to be context-dependent, influenced by factors such as MP aging, biofilm formation, particle size, polymer type, and release kinetics in digestive fluids or intracellular environments 44, 51. Under current evidence, the Trojan horse effect should not be regarded as an established exposure mechanism for phenol. Instead, it is more appropriately considered a context-dependent and mechanistically plausible hypothesis, in which MPs may modify the local bioavailability and downstream signaling responses of phenol under specific environmental and biological conditions 46, 47, 56.

Overall, while the Trojan horse effect has been supported in co-exposure studies involving hydrophobic phenolic compounds, current evidence suggests that its applicability to phenol remains insufficiently validated under environmentally relevant exposure conditions.

In the following sections, this hypothesis is used as a conceptual framework to link MP-induced oxidative stress with phenol-related signaling pathways, and to evaluate their potential roles in tumor-related endpoints and microenvironmental processes, thereby helping to define key priorities for future co-exposure research. Additional studies directly examining phenol adsorption, transport efficiency, and intracellular release from MPs are needed before the environmental relevance of this mechanism can be established.

## 5. Potential roles of microplastics in modulating phenolic exposure within the tumor microenvironment

The tumor microenvironment (TME) is a highly dynamic and heterogeneous biological network composed of tumor cells, immune cells, stromal cells, extracellular matrix, and soluble factors that collectively regulate tumor initiation, progression, immune evasion, and therapeutic response 57, 58. Notably, key features of the TME, including chronic inflammation, metabolic reprogramming, abnormal angiogenesis, and immunosuppression, are closely linked to cellular redox status and redox-associated signaling pathways 58-60. Recent studies suggest that MPs may induce persistent intracellular reactive oxygen species (ROS) production through mitochondrial dysfunction and redox imbalance 61, 62. Recent evidence further indicates that MPs have been identified in multiple tumor types, including lung, gastric, breast, and prostate cancers, and may remain intracellularly persistent owing to their resistance to enzymatic degradation 63, 64**.** In tumor tissues, such persistence may contribute to a sustained oxidative and metabolic burden, thereby reinforcing mitochondrial dysfunction and redox imbalance over time 65, 66. In addition, MPs can be internalized by immune and epithelial cells via endocytosis, leading to metabolic disruption and the release of pro-inflammatory cytokines 67-69. However, whether distinct internalization routes, such as macropinocytosis, clathrin-mediated endocytosis, or direct membrane penetration, result in differential intracellular trafficking, subcellular localization, or retention of MPs remains poorly understood. Furthermore, the extent to which these uptake pathways influence MP distribution and persistence within tumor tissues under physiologically relevant conditions has not been systematically investigated. Addressing these questions through clinical specimens and tumor-bearing models will be important for clarifying the biological significance of MP accumulation within the tumor microenvironment.

Under these conditions, the sustained intracellular presence of MPs within tumor tissues may establish a persistent redox-active background that interacts with the pre-existing oxidative milieu of the TME. These observations suggest that MPs may influence local redox conditions within the TME, although the extent to which such effects contribute to downstream signaling remains incompletely understood. The mechanistic implications of this redox interaction for downstream signaling and tumor microenvironment remodeling are examined in Section 6.

## 6. MPs-mediated phenolic exposure and redox-sensitive signaling: MAPK as the central convergence framework

**Figure 2:**
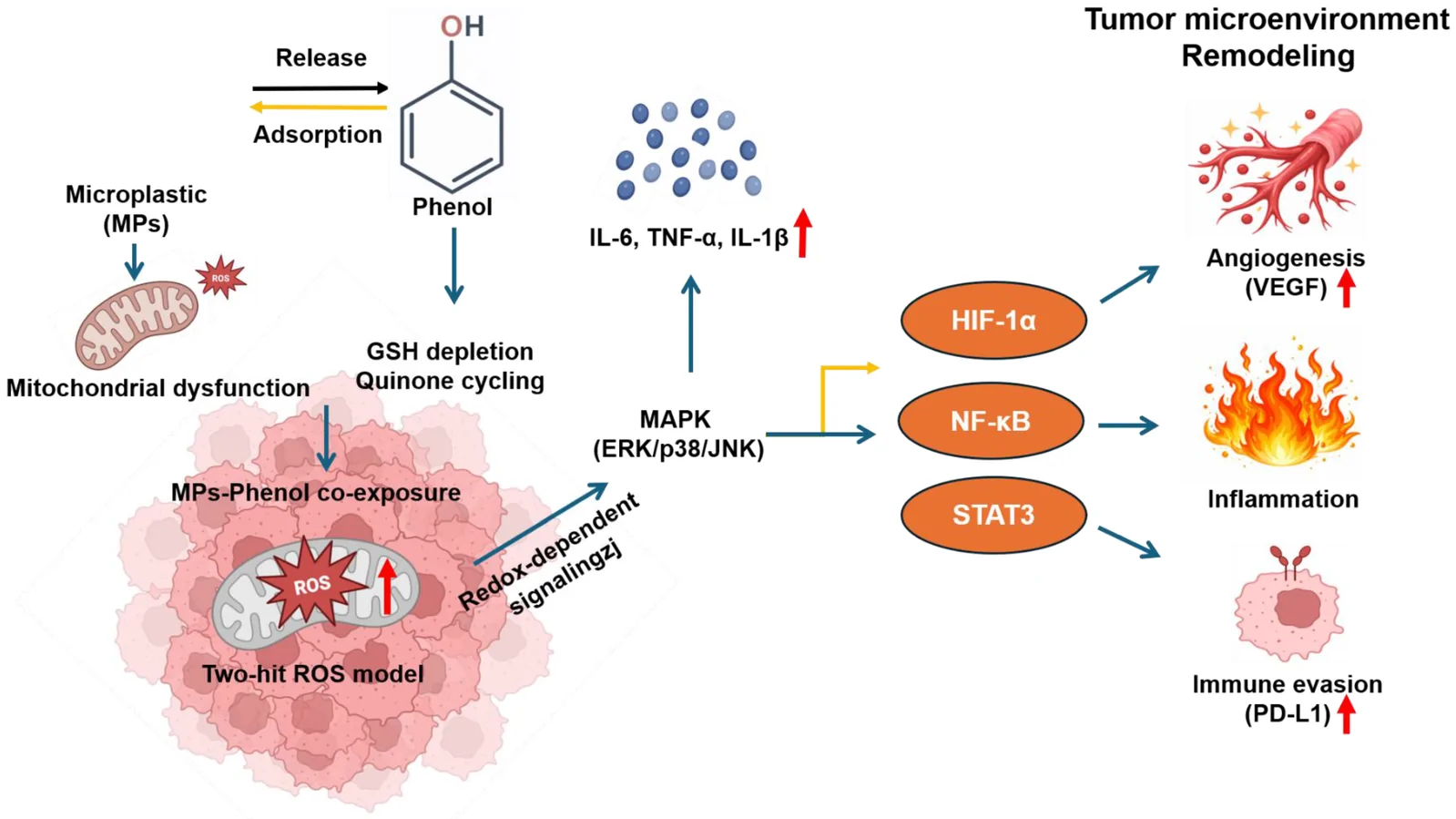
** Proposed framework of MPs-phenol co-exposure in tumor microenvironment remodeling.** Microplastics (MPs) may induce mitochondrial dysfunction and ROS production, while phenol disrupts antioxidant systems via GSH depletion and quinone cycling. These distinct mechanisms may jointly contribute to a two-hit oxidative stress model. The resulting redox imbalance may influence MAPK signaling pathways (ERK, p38, JNK), which in turn may affect downstream transcription factors, including HIF-1α, NF-κB, and STAT3. These signaling cascades may contribute to tumor-associated processes such as angiogenesis, inflammation, and immune evasion, thereby potentially influencing tumor microenvironment remodeling. The proposed framework integrates direct experimental evidence and mechanistic inference and should therefore be interpreted as a testable hypothesis rather than a confirmed causal pathway.

Based on the redox-active properties of MPs within the TME discussed above, Fig. 2 presents a conceptual framework illustrating how MPs-phenol co-exposure may jointly contribute to intracellular oxidative stress and potentially influence downstream tumor-associated signaling pathways. The mechanistic basis of this framework is examined in the following sections.

### 6.1 MAPK convergence

Building upon the redox-active properties of MPs within the TME outlined above, MP-induced oxidative stress and phenol-mediated antioxidant disruption may jointly contribute to intracellular redox imbalance under co-exposure conditions.

Importantly, these effects may arise through mechanistically non-overlapping pathways: phenol primarily depletes reduced glutathione (GSH) via quinone intermediates and impairs antioxidant enzyme function 70, 71, whereas MPs mainly induce ROS through mitochondrial electron transport chain uncoupling and increased lysosomal membrane permeability 66, 72. In addition, nanoscale particles generally exhibit greater ROS-inducing efficiency than microscale particles because of enhanced cellular penetration and mitochondrial interference 73. Together, these mechanistic differences support a potential two-hit stress model in which particle-mediated mitochondrial stress and chemically induced antioxidant disruption may jointly contribute to aberrant redox-sensitive signaling regulation.

Within this framework, the MAPK family may function as a canonical redox-sensitive pathway and a potential convergence node integrating redox signals induced by particulate and chemical exposures 74, 75. Its branches exhibit distinct roles: extracellular signal-regulated kinase (ERK) is typically linked to proliferation and survival, whereas p38 and c-Jun N-terminal kinase (JNK) are more closely associated with oxidative stress responses, inflammatory signaling, and context-dependent cell fate regulation 76. This functional divergence suggests that, under MPs-phenol co-exposure-associated redox imbalance, MAPK signaling is unlikely to be uniformly activated and may instead show branch-selective activation depending on the source, intensity, and duration of oxidative stress. Accumulating evidence supports ROS-mediated regulation of MAPK pathways in response to MPs and several structurally related phenolic compounds. However, direct evidence demonstrating MAPK activation under MPs-phenol co-exposure conditions remains limited 77, 78. For example, polystyrene nanoplastics (PS-NPs) activate p38 or JNK signaling in cellular and *in vivo* models through ROS accumulation, accompanied by ferroptosis, inflammatory responses, mitochondrial dysfunction, and endoplasmic reticulum stress 79-81. Likewise, several structurally related phenolic compounds have been shown to induce differential activation of p38, JNK, or ERK through ROS-dependent mechanisms. Although these observations are derived from phenolic analogs rather than phenol itself, they suggest that variations in ROS source and intensity may influence MAPK branch specificity 70, 82. Importantly, recent studies have shown that phenol activates ERK/p38 signaling in both cellular and zebrafish models, providing direct mechanistic support for the phenol-MAPK axis 83.

Taken together, these findings suggest that MAPK may represent a potential convergence node through which redox imbalance potentially associated with MPs-phenol co-exposure could influence downstream transcriptional responses in a branch-selective and context-dependent manner, with implications for tumor-associated signaling discussed in the following section.

### 6.2 Potential MAPK-associated angiogenic and metastatic reprogramming

In the TME, hypoxia-inducible factor 1α (HIF-1α) is widely regarded as a core transcriptional regulator that integrates hypoxic, oxidative, and metabolic signals 84. Notably, HIF-1α activation is not limited to traditional hypoxic conditions. Under normoxic conditions, sustained activation of upstream signaling pathways (e.g., MAPK) can induce a phenomenon known as “pseudohypoxia,” leading to abnormal HIF-1α stabilization and activation of related transcriptional programs 85, 86.

At the molecular level, sustained ERK/p38 phosphorylation may enhance HIF-1α expression and activity. This process may involve inhibition of prolyl hydroxylase domain enzymes (PHDs), thereby reducing ubiquitination and proteasomal degradation 87-89. Through the MAPK-HIF-1α axis, upstream redox imbalance can be translated into transcriptional outputs that may contribute to tumor-associated processes. Once stabilized, HIF-1α directly binds to hypoxia response elements (HREs) in the promoters of target genes such as vascular endothelial growth factor (VEGF), promoting their transcription and potentially contributing to tumor-associated angiogenesis 84, 90. This process stimulates endothelial cell proliferation, migration, and tube formation, ultimately contributing to the formation of a structurally abnormal yet functional vascular network that supports tumor growth and facilitates tumor cell dissemination. Sustained HIF-1α activation is also closely associated with EMT-related transcriptional reprogramming, thereby enhancing tumor cell invasion and metastatic potential. HIF-1α may induce core EMT transcription factors such as Snail, leading to downregulation of epithelial markers (e.g., E-cadherin) and upregulation of mesenchymal markers (e.g., N-cadherin and Vimentin), ultimately increasing cell motility and invasiveness 91.

Notably, MPs and NPs are not merely passive exposure backgrounds or carriers; they may also directly induce phenotypic changes associated with migration and invasion 74. In both human bronchial and alveolar epithelial cells and breast epithelial and cancer cell models, polyethylene and polystyrene micro/nanoplastics have been shown to induce EMT, cytoskeletal reorganization, and increased proliferative and migratory potential, even in the absence of significant cytotoxicity 92, 93. Collectively, these studies suggest that MPs may not only provide a persistent oxidative stress background but also potentially shift cells toward a more migratory and invasive state, thereby increasing the biological plausibility that tumor-related phenotypes may be enhanced or altered under co-exposure conditions 94, 95.

Beyond angiogenesis and metastasis, MAPK signaling may also extend to inflammatory and immune-related transcriptional networks. Redox-sensitive pathways such as NF-κB and STAT3 interact with MAPK signaling and play important roles in shaping the tumor immune microenvironment by promoting the expression of pro-inflammatory cytokines (e.g., IL-6, TNF-α) and immunoregulatory factors 96, 97. In addition, both NF-κB and STAT3 are implicated in the transcriptional regulation of immune checkpoint molecules such as PD-L1, suggesting a potential mechanistic link between redox signaling and tumor immune evasion 98, 99.

In co-exposure settings, MPs may provide a persistent intracellular ROS-associated background that could influence redox-sensitive transcriptional programs 100. Under such conditions, MPs-phenol co-exposure may contribute to sustained or altered redox-sensitive signaling, thereby potentially affecting inflammatory, angiogenic, and immune-regulatory responses. Taken together, existing evidence suggests that MAPK may serve as a potential hub that translates redox imbalance into integrated transcriptional reprogramming across angiogenesis, metastasis, and immune regulation 84. The reported direct effects of MPs/NPs on EMT, cell migration, and tumor-promoting phenotypes further strengthen the biological rationale that MPs-phenol co-exposure may enhance or contribute to downstream tumor-related outputs 74, 94. However, direct experimental evidence supporting these integrated effects remains limited, and this model therefore should be interpreted as a biologically plausible and testable hypothesis rather than a confirmed causal mechanism. Further validation will require co-exposure *in vivo* systems, time-series signaling analyses, and functional phenotypic measurements 95. To further clarify the evidence hierarchy supporting this framework, Table 2 summarizes the current evidence for MPs, phenol, phenolic analogs, and MPs-phenol co-exposure across key biological processes.

## 7. Conclusion and future perspectives

Building upon the two-hit oxidative stress model and MAPK-centered signaling framework proposed herein, this review positions MPs-phenol co-exposure as a biologically plausible yet experimentally underexplored context that may influence tumor microenvironment dynamics, with potential implications for angiogenesis, chronic inflammation, and immune evasion.

Notably, although phenol is widely used and environmentally prevalent, it has received comparatively less attention in tumor-related environmental toxicology than well-characterized pollutants such as BPA and polycyclic aromatic hydrocarbons (PAHs) 10, 101. Compared with more extensively studied hydrophobic phenolic derivatives, phenol exhibits higher water solubility and environmental mobility, suggesting that its behavior and biological effects under co-exposure conditions may differ; however, these aspects remain insufficiently explored 19, 20. Accordingly, this review highlights phenol as a potentially underrecognized modulator of redox-sensitive signaling, particularly under conditions in which it coexists with particulate contaminants such as microplastics, where its biological effects may be further modified. Despite the integrative framework proposed here, direct experimental evidence linking MPs-phenol co-exposure to coordinated signaling outcomes and tumor-associated phenotypes remains limited. Existing studies predominantly focus on single-pollutant exposure and lack systematic evaluation under environmentally relevant concentrations, chronic low-dose conditions, and co-exposure scenarios 3-5. Furthermore, direct clinical evidence linking MPs-phenol co-exposure with immunotherapeutic response or patient prognosis is currently unavailable. Although MPs have been detected in human tumor tissues and biological samples, the clinical significance of their interaction with phenol remains unclear. Future epidemiological, translational, and clinical studies will be required to determine whether such co-exposures influence disease progression, therapeutic response, or patient outcomes. In addition, microplastic properties, including particle size, aging status, surface characteristics, and interactions with coexisting pollutants may critically influence their biological effects, yet the underlying mechanisms remain poorly defined 40, 41, 43.

Future studies should focus on developing integrated experimental models that more accurately reflect real-world environmental conditions, including co-exposure systems, chronic low-dose designs, and *in vivo* validation platforms, to systematically assess the interactions between MPs and chemical pollutants and their effects on cellular signaling. Furthermore, integrating multi-omics approaches, temporal dynamic analyses, and dose-response studies will be essential for elucidating interactions among different pollutants and their impacts on signaling pathways, cellular functions, and the tumor microenvironment. Incorporating microplastic aging processes and surface characteristics into experimental design will also improve the environmental relevance of exposure models. Addressing these critical knowledge gaps will advance our understanding of the biological effects of complex pollutant co-exposures and provide a more realistic, mechanistically informed basis for environmental risk assessment and public health decision-making.

## Figures and Tables

**Table 1:** Human-relevant exposure routes of microplastics, size distributions, polymer composition, and potential for biological internalization

Exposure route	Typical size range	Representative	Dominant polymers	Cellular uptake relevance	Reference
Drinking water (tap & bottled)	50-600 nm; 1-100 μm (majority < 50 μm)	Tap: ~10³ particles/L; Bottled: 10³-10⁴ particles/L	PET, PP, PE, PS	1-10 μm internalized; < 1 μm may cross gut barrier	65, 78
Seafood	10-500 μm; shellfish 100-200 μm; nano-MPs < 100 nm	Fish: 1-6/ind.; Shellfish: 0.1-0.3 MPs/g (up to 55/ind.); Crustaceans: 0.05-0.09 MPs/g	PS, PP, PE, PET, PA/Nylon, PVC	Shellfish < 150 μm consumed whole; < 10 μm interact with gut epithelium	102-104
Indoor air / household dust	10-500 μm; inhalable < 10 μm; respirable < 5 μm; NPs < 100 nm	Indoor air: 4,000-14,000 particles/m³; Inhalation: 2-150 MPs/kg-bw/day (adults), up to ~7,900 (infants)	Polyester/PA fibers, PE, PP, PS, PVC, PVB	Small MPs/NPs reach bronchioles & alveoli	105, 106
Human blood	Predominantly 10-100 μm; fragments < 50 μm most abundant	Healthy adults: 4.2 MPs/mL (88.9% detection); Thrombi: 14.9-234 μg/g	PS, PP, PE, PET, PA	Entry via GI, inhalation, medical sources	107, 108

Abbreviations: PET, polyethylene terephthalate; PP, polypropylene; PE, polyethylene; PS, polystyrene; PA, polyamide; PVC, polyvinyl chloride; PVB, polyvinyl butyral.

**Table 2:** Summary of current evidence related to the proposed MPs-phenol co-exposure framework.

Biological process	MPs	Phenol	Phenolic analogs	MPs-Phenol co-exposure	Reference
Trojan horse effect	Supported	Limited evidence	Supported	Limited direct evidence	30, 44-48, 50-55
ROS generation	Supported	Supported	Supported	Limited direct evidence	61, 62, 66, 70, 71, 78, 81, 94, 109
MAPK activation	Supported	Supported	Supported	Not directly investigated	61, 66, 74, 76, 78, 80-83
NF-κB / STAT3 signaling	Supported	Limited evidence	Limited evidence	Not directly investigated	94, 95, 100
HIF-1α / VEGF signaling	Limited evidence	Supported	Limited evidence	Not directly investigated	83, 85, 87-89
EMT reprogramming	Supported	Supported	Supported	Not directly investigated	71, 78, 80, 92, 93
PD-L1 regulation and immune evasion	Limited evidence	Limited evidence	Limited evidence	Not directly investigated	97, 100
TME remodeling	Limited evidence	Limited evidence	Limited evidence	Proposed hypothesis	57-59, 94, 95

## Data Availability

The original contributions presented in the study are included in the article/supplementary material. Further inquiries can be directed to the corresponding author.

## References

[1] Li W, Zu B, Yang Q, Guo J, Li J (2023). Sources, distribution, and environmental effects of microplastics: a systematic review. RSC Adv.

[2] Osman AI, Hosny M, Eltaweil AS, Omar S, Elgarahy AM, Farghali M (2023). Microplastic sources, formation, toxicity and remediation: a review. Environ Chem Lett.

[3] Drakvik E, Altenburger R, Aoki Y, Backhaus T, Bahadori T, Barouki R (2020). Statement on advancing the assessment of chemical mixtures and their risks for human health and the environment. Environ Int.

[4] Quiros-Alcala L, Barr DB (2023). Invited Perspective: Mixtures-Are They Worth the Risk (Assessment)?. Environ Health Perspect.

[5] Rager JE, Rider CV (2023). Wrangling Whole Mixtures Risk Assessment: Recent Advances in Determining Sufficient Similarity. Curr Opin Toxicol.

[6] Li Y, Tao L, Wang Q, Wang F, Li G, Song M (2023). Potential Health Impact of Microplastics: A Review of Environmental Distribution, Human Exposure, and Toxic Effects. Environ Health (Wash).

[7] Li C, Gillings MR, Zhang C, Chen Q, Zhu D, Wang J (2024). Ecology and risks of the global plastisphere as a newly expanding microbial habitat. Innovation (Camb).

[8] Cássio F, Batista D, Pradhan A (2022). Plastic Interactions with Pollutants and Consequences to Aquatic Ecosystems: What We Know and What We Do Not Know. Biomolecules.

[9] Sørensen L, Rogers E, Altin D, Salaberria I, Booth AM (2020). Sorption of PAHs to microplastic and their bioavailability and toxicity to marine copepods under co-exposure conditions. Environ Pollut.

[10] Rubinstein J, Pinney SM, Xie C, Wang HS (2024). Association of same-day urinary phenol levels and cardiac electrical alterations: analysis of the Fernald Community Cohort. Environ Health.

[11] Yan J, Meng Q, Shen X, Chen B, Sun Y, Xiang J (2020). Selective valorization of lignin to phenol by direct transformation of C(sp2)-C(sp3) and C-O bonds. Sci Adv.

[12] Bih NL, Rwiza MJ, Ripanda AS, Mahamat AA, Machunda RL, Choi JW (2025). Adsorption of phenol and methylene blue contaminants onto high-performance catalytic activated carbon from biomass residues. Heliyon.

[13] Ahrens A, Bonde A, Sun H, Wittig NK, Hammershøj HCD, Batista GMF (2023). Catalytic disconnection of C-O bonds in epoxy resins and composites. Nature.

[14] Moualhi A, Rios De Anda A, Vahabi H, Chatti S, Abderrazak H, Renard E (2023). Toward Sustainable Phenolic Resins from Biobased Aldehydes Using Spark Plasma Sintering. ACS Sustainable Chemistry & Engineering.

[15] Cathey AL, Nguyen VK, Colacino JA, Woodruff TJ, Reynolds P, Aung MT (2023). Exploratory profiles of phenols, parabens, and per- and poly-fluoroalkyl substances among NHANES study participants in association with previous cancer diagnoses. J Expo Sci Environ Epidemiol.

[16] Parada H Jr, Gammon MD, Ettore HL, Chen J, Calafat AM, Neugut AI (2019). Urinary concentrations of environmental phenols and their associations with breast cancer incidence and mortality following breast cancer. Environ Int.

[17] Grace Pavithra K, Sundar Rajan P, Arun J, Brindhadevi K, Hoang Le Q, Pugazhendhi A (2023). A review on recent advancements in extraction, removal and recovery of phenols from phenolic wastewater: Challenges and future outlook. Environ Res.

[18] Kamal SK, Mustafa ZM, Abbas AS (2025). Reaction Kinetics and Mass Transfer of Photocatalytic Fenton for Phenol Degradation in a Petroleum Refinery Wastewater. Tikrit Journal of Engineering Sciences.

[19] Khan MJ, Wibowo A, Karim Z, Posoknistakul P, Matsagar BM, Wu KC (2024). Wastewater Treatment Using Membrane Bioreactor Technologies: Removal of Phenolic Contaminants from Oil and Coal Refineries and Pharmaceutical Industries. Polymers (Basel).

[20] Xue YX, Dai FF, Yang Q, Chen JH, Lin QJ, Fang LJ (2022). Fabrication of PEBA/HZIF-8 Pervaporation Membranes for High Efficiency Phenol Recovery. ACS Omega.

[21] Tumu K, Vorst K, Curtzwiler G (2023). Endocrine modulating chemicals in food packaging: A review of phthalates and bisphenols. Compr Rev Food Sci Food Saf.

[22] Yaqoob AA, Al-Zaqri N, Alamzeb M, Hussain F, Oh SE, Umar K (2023). Bioenergy Generation and Phenol Degradation through Microbial Fuel Cells Energized by Domestic Organic Waste. Molecules.

[23] Fadeel B, Alexander J, Antunes SC, Dalhoff K, Fritsche E, Hogberg HT (2024). Editorial: Five grand challenges in toxicology. Front Toxicol.

[24] Schmeisser S, Miccoli A, von Bergen M, Berggren E, Braeuning A, Busch W (2023). New approach methodologies in human regulatory toxicology - Not if, but how and when!. Environ Int.

[25] Smirnova L, Kleinstreuer N, Corvi R, Levchenko A, Fitzpatrick SC, Hartung T (2018). 3S - Systematic, systemic, and systems biology and toxicology. Altex.

[26] Honza T, El Yamani N, Dusinska M, Rundén-Pran E, Marcon F (2024). New advanced models (NAMs) for risk assessment of bisphenol A alternatives. Efsa j.

[27] Maertens A, Golden E, Hartung T (2021). Avoiding Regrettable Substitutions: Green Toxicology for Sustainable Chemistry. ACS Sustain Chem Eng.

[28] Qadeer A, Anis M, Warner GR, Potts C, Giovanoulis G, Nasr S (2024). Global Environmental and Toxicological Data of Emerging Plasticizers: Current Knowledge, Regrettable Substitution Dilemma, Green Solution and Future Perspectives. Green Chem.

[29] Alva-Gallegos R, Jirkovský E, Mladěnka P, Carazo A (2024). Small phenolic compounds as potential endocrine disruptors interacting with estrogen receptor alpha. Front Endocrinol (Lausanne).

[30] Long F, Ren Y, Bi F, Wu Z, Zhang H, Li J (2025). Contamination Characterization, Toxicological Properties, and Health Risk Assessment of Bisphenols in Multiple Media: Current Research Status and Future Perspectives. Toxics.

[31] Xiong Y, Li Z, Xiong X, Luo Z, Zhong K, Hu J (2025). Associations between phenol and paraben exposure and the risk of developing breast cancer in adult women: a cross-sectional study. Sci Rep.

[32] Kehm RD, Lloyd SE, Burke KR, Terry MB (2025). Advancing environmental epidemiologic methods to confront the cancer burden. Am J Epidemiol.

[33] Thompson RC, Courtene-Jones W, Boucher J, Pahl S, Raubenheimer K, Koelmans AA (2024). Twenty years of microplastic pollution research-what have we learned?. Science.

[34] Zhao B, Richardson RE, You F (2024). Microplastics monitoring in freshwater systems: A review of global efforts, knowledge gaps, and research priorities. J Hazard Mater.

[35] Giustra M, Sinesi G, Spena F, De Santes B, Morelli L, Barbieri L (2024). Microplastics in Cosmetics: Open Questions and Sustainable Opportunities. ChemSusChem.

[36] En-Nejmy K, El Hayany B, Al-Alawi M, Jemo M, Hafidi M, El Fels L (2024). Microplastics in soil: A comprehensive review of occurrence, sources, fate, analytical techniques and potential impacts. Ecotoxicol Environ Saf.

[37] Waldman WR, Rillig MC (2020). Microplastic Research Should Embrace the Complexity of Secondary Particles. Environ Sci Technol.

[38] Landrigan PJ, Stegeman JJ, Fleming LE, Allemand D, Anderson DM, Backer LC (2020). Human Health and Ocean Pollution. Ann Glob Health.

[39] Zhao S, Kvale KF, Zhu L, Zettler ER, Egger M, Mincer TJ (2025). The distribution of subsurface microplastics in the ocean. Nature.

[40] Lu Q, Zhou Y, Sui Q, Zhou Y (2023). Mechanism and characterization of microplastic aging process: A review. Front Environ Sci Eng.

[41] Luo H, Liu C, He D, Xu J, Sun J, Li J (2022). Environmental behaviors of microplastics in aquatic systems: A systematic review on degradation, adsorption, toxicity and biofilm under aging conditions. J Hazard Mater.

[42] Luo H, Liu C, He D, Sun J, Li J, Pan X (2022). Effects of aging on environmental behavior of plastic additives: Migration, leaching, and ecotoxicity. Sci Total Environ.

[43] Luo H, Tu C, He D, Zhang A, Sun J, Li J (2023). Interactions between microplastics and contaminants: A review focusing on the effect of aging process. Sci Total Environ.

[44] Hu L, Zhao Y, Xu H (2022). Trojan horse in the intestine: A review on the biotoxicity of microplastics combined environmental contaminants. J Hazard Mater.

[45] Zhao W, Zhao Y, Geng T, Tian Y, Zhao P (2023). Co-transport behavior and Trojan-horse effect of colloidal microplastics with different functional groups and heavy metals in porous media. J Hazard Mater.

[46] Yan Y, Cheng J, Gao J, Liu Y, Tian H, Liu Y (2025). Exploring Environmental Behaviors and Health Impacts of Biodegradable Microplastics. Environ Sci Technol.

[47] Yang H, Chen Z, Kong L, Xing H, Yang Q, Wu J (2025). A Review of Eco-Corona Formation on Micro/Nanoplastics and Its Effects on Stability, Bioavailability, and Toxicity. Water.

[48] Ateia M, Ersan G, Alalm MG, Boffito DC, Karanfil T (2022). Emerging investigator series: microplastic sources, fate, toxicity, detection, and interactions with micropollutants in aquatic ecosystems - a review of reviews. Environ Sci Process Impacts.

[49] Carvalho JGRd, Augusto HC, Ferraz R, Delerue-Matos C, Fernandes VC (2024). Micro(nano)plastic and Related Chemicals: Emerging Contaminants in Environment, Food and Health Impacts. Toxics.

[50] He L, Zhuang WE, Hur J, Yang L (2026). Interactions of microplastics, dissolved organic matter, and coexisting pollutants: Mechanisms, environmental implications, and knowledge gaps. Environ Res.

[51] Prus Z, Styszko K (2025). Wastewater-Derived Microplastics as Carriers of Aromatic Organic Contaminants (AOCs): A Critical Review of Ageing, Sorption Mechanisms, and Environmental Implications. Int J Mol Sci.

[52] Wei X, Li M, Wang Y, Jin L, Ma G, Yu H (2019). Developing Predictive Models for Carrying Ability of Micro-Plastics towards Organic Pollutants. Molecules.

[53] Ding F, Wang H, Li Y, Leng X, Gao J, Huang D (2024). Polystyrene microplastics with absorbed nonylphenol induce intestinal dysfunction in human Caco-2 cells. Environ Toxicol Pharmacol.

[54] Ye T, Yang R, He S, Li J, Liu Y, Li C (2025). Synergistic endocrine disruption and cellular toxicity of polyethylene microplastics and bisphenol A in MLTC-1 cells and zebrafish. Sci Rep.

[55] Xia Y, Niu S, Yu J (2023). Microplastics as vectors of organic pollutants in aquatic environment: A review on mechanisms, numerical models, and influencing factors. Sci Total Environ.

[56] Bastante-Rabadán M, Boltes K (2024). Mixtures of Micro and Nanoplastics and Contaminants of Emerging Concern in Environment: What We Know about Their Toxicological Effects. Toxics.

[57] Ugai T, Yao Q, Ugai S, Ogino S (2024). Advancing precision oncology: Insights into the tumor microenvironment and immunotherapy outcomes. Innovation (Camb).

[58] Goenka A, Khan F, Verma B, Sinha P, Dmello CC, Jogalekar MP (2023). Tumor microenvironment signaling and therapeutics in cancer progression. Cancer Commun (Lond).

[59] Iqbal MJ, Kabeer A, Abbas Z, Siddiqui HA, Calina D, Sharifi-Rad J (2024). Interplay of oxidative stress, cellular communication and signaling pathways in cancer. Cell Commun Signal.

[60] Liu W, Wang B, Zhou M, Liu D, Chen F, Zhao X (2023). Redox Dysregulation in the Tumor Microenvironment Contributes to Cancer Metastasis. Antioxid Redox Signal.

[61] Liu T, Hou B, Wang Z, Yang Y (2022). Polystyrene microplastics induce mitochondrial damage in mouse GC-2 cells. Ecotoxicol Environ Saf.

[62] Zeng G, Li J, Wang Y, Su J, Lu Z, Zhang F (2024). Polystyrene microplastic-induced oxidative stress triggers intestinal barrier dysfunction via the NF-κB/NLRP3/IL-1β/MCLK pathway. Environ Pollut.

[63] Gałęziewska J, Gromek P, Kruczkowska W, Grabowska K, Jęckowski M, Capuano F (2025). Evaluating the relationship between microplastics and nanoplastics contamination and diverse cancer types development. Environ Pollut.

[64] Mishra SK, Sanyal T, Kundu P, Kumar R, Ghosh D, Chakrabarti G (2025). Microplastics as emerging carcinogens: from environmental pollutants to oncogenic drivers. Mol Cancer.

[65] Kadac-Czapska K, Ośko J, Knez E, Grembecka M (2024). Microplastics and Oxidative Stress-Current Problems and Prospects. Antioxidants (Basel).

[66] Lee SE, Yi Y, Moon S, Yoon H, Park YS (2022). Impact of Micro- and Nanoplastics on Mitochondria. Metabolites.

[67] Hamza A, Ijaz MU, Anwar H (2023). Rhamnetin alleviates polystyrene microplastics-induced testicular damage by restoring biochemical, steroidogenic, hormonal, apoptotic, inflammatory, spermatogenic and histological profile in male albino rats. Hum Exp Toxicol.

[68] Rizwan A, Ijaz MU, Hamza A, Anwar H (2023). Attenuative effect of astilbin on polystyrene microplastics induced testicular damage: Biochemical, spermatological and histopathological-based evidences. Toxicol Appl Pharmacol.

[69] Wolff CM, Singer D, Schmidt A, Bekeschus S (2023). Immune and inflammatory responses of human macrophages, dendritic cells, and T-cells in presence of micro- and nanoplastic of different types and sizes. J Hazard Mater.

[70] Bolton JL, Dunlap T (2017). Formation and Biological Targets of Quinones: Cytotoxic versus Cytoprotective Effects. Chem Res Toxicol.

[71] Tolosa L, Martínez-Sena T, Schimming JP, Moro E, Escher SE, Ter Braak B (2021). The *in vitro* assessment of the toxicity of volatile, oxidisable, redox-cycling compounds: phenols as an example. Arch Toxicol.

[72] Han SW, Choi J, Ryu KY (2024). Recent progress and future directions of the research on nanoplastic-induced neurotoxicity. Neural Regen Res.

[73] Płuciennik K, Sicińska P, Misztal W, Bukowska B (2024). Important Factors Affecting Induction of Cell Death, Oxidative Stress and DNA Damage by Nano- and Microplastic Particles *In Vitro*. Cells.

[74] Chen Y, Zhang Z, Ji K, Zhang Q, Qian L, Yang C (2025). Role of microplastics in the tumor microenvironment (Review). Oncol Lett.

[75] Franci L, Vallini G, Bertolino FM, Cicaloni V, Inzalaco G, Cicogni M (2024). MAPK15 controls cellular responses to oxidative stress by regulating NRF2 activity and expression of its downstream target genes. Redox Biol.

[76] Burgermeister E (2023). Mitogen-Activated Protein Kinase and Exploratory Nuclear Receptor Crosstalk in Cancer Immunotherapy. Int J Mol Sci.

[77] Lim EY, Kim GD (2024). Particulate Matter-Induced Emerging Health Effects Associated with Oxidative Stress and Inflammation. Antioxidants (Basel).

[78] Mahmud F, Sarker DB, Jocelyn JA, Sang QA (2024). Molecular and Cellular Effects of Microplastics and Nanoplastics: Focus on Inflammation and Senescence. Cells.

[79] Fan J, Liu L, Lu Y, Chen Q, Fan S, Yang Y (2024). Acute exposure to polystyrene nanoparticles promotes liver injury by inducing mitochondrial ROS-dependent necroptosis and augmenting macrophage-hepatocyte crosstalk. Part Fibre Toxicol.

[80] He Y, Yu T, Li H, Sun Q, Chen M, Lin Y (2024). Polystyrene nanoplastic exposure actives ferroptosis by oxidative stress-induced lipid peroxidation in porcine oocytes during maturation. J Anim Sci Biotechnol.

[81] Khan A, Jia Z (2023). Recent insights into uptake, toxicity, and molecular targets of microplastics and nanoplastics relevant to human health impacts. iScience.

[82] Fang T, Huang YK, Wei J, Monterrosa Mena JE, Lakey PSJ, Kleinman MT (2022). Superoxide Release by Macrophages through NADPH Oxidase Activation Dominating Chemistry by Isoprene Secondary Organic Aerosols and Quinones to Cause Oxidative Damage on Membranes. Environ Sci Technol.

[83] Lin CY, Liu W, Chen PH, Wang CC, Lee CH (2026). Phenol exposure promotes tumor-related signaling and blood vessel formation through the extracellular signal-regulated kinase/p38/hypoxia-inducible factor-1α pathway in cellular and zebrafish models. Comp Biochem Physiol C Toxicol Pharmacol.

[84] Wicks EE, Semenza GL (2022). Hypoxia-inducible factors: cancer progression and clinical translation. J Clin Invest.

[85] Hayashi Y, Yokota A, Harada H, Huang G (2019). Hypoxia/pseudohypoxia-mediated activation of hypoxia-inducible factor-1α in cancer. Cancer Sci.

[86] Sang N, Stiehl DP, Bohensky J, Leshchinsky I, Srinivas V, Caro J (2003). MAPK signaling up-regulates the activity of hypoxia-inducible factors by its effects on p300. J Biol Chem.

[87] Gaete D, Rodriguez D, Watts D, Sormendi S, Chavakis T, Wielockx B (2021). HIF-Prolyl Hydroxylase Domain Proteins (PHDs) in Cancer-Potential Targets for Anti-Tumor Therapy?. Cancers (Basel).

[88] Ivan M, Kondo K, Yang H, Kim W, Valiando J, Ohh M (2001). HIFalpha targeted for VHL-mediated destruction by proline hydroxylation: implications for O2 sensing. Science.

[89] Jaakkola P, Mole DR, Tian YM, Wilson MI, Gielbert J, Gaskell SJ (2001). Targeting of HIF-alpha to the von Hippel-Lindau ubiquitylation complex by O2-regulated prolyl hydroxylation. Science.

[90] Basheeruddin M, Qausain S (2024). Hypoxia-Inducible Factor 1-Alpha (HIF-1α) and Cancer: Mechanisms of Tumor Hypoxia and Therapeutic Targeting. Cureus.

[91] Tam SY, Wu VWC, Law HKW (2020). Hypoxia-Induced Epithelial-Mesenchymal Transition in Cancers: HIF-1α and Beyond. Front Oncol.

[92] Traversa A, Mari E, Pontecorvi P, Gerini G, Romano E, Megiorni F (2024). Polyethylene Micro/Nanoplastics Exposure Induces Epithelial-Mesenchymal Transition in Human Bronchial and Alveolar Epithelial Cells. Int J Mol Sci.

[93] Schnee M, Sieler M, Dörnen J, Dittmar T (2024). Effects of polystyrene nano- and microplastics on human breast epithelial cells and human breast cancer cells. Heliyon.

[94] Cheng Y, Yang Y, Bai L, Cui J (2024). Microplastics: an often-overlooked issue in the transition from chronic inflammation to cancer. J Transl Med.

[95] Deng X, Gui Y, Zhao L (2025). The micro(nano)plastics perspective: exploring cancer development and therapy. Mol Cancer.

[96] Grivennikov SI, Greten FR, Karin M (2010). Immunity, inflammation, and cancer. Cell.

[97] Shrihari TG (2017). Dual role of inflammatory mediators in cancer. Ecancermedicalscience.

[98] Antonangeli F, Natalini A, Garassino MC, Sica A, Santoni A, Di Rosa F (2020). Regulation of PD-L1 Expression by NF-κB in Cancer. Front Immunol.

[99] Wang Y, Chen Y, Liu Y, Zhao J, Wang G, Chen H (2025). Tumor vascular endothelial cells promote immune escape by upregulating PD-L1 expression via crosstalk between NF-κB and STAT3 signaling pathways in nasopharyngeal carcinoma. Cell Death Dis.

[100] Domenech J, Annangi B, Marcos R, Hernández A, Catalán J (2023). Insights into the potential carcinogenicity of micro- and nano-plastics. Mutat Res Rev Mutat Res.

[101] Della Rocca Y, Traini EM, Diomede F, Fonticoli L, Trubiani O, Paganelli A (2023). Current Evidence on Bisphenol A Exposure and the Molecular Mechanism Involved in Related Pathological Conditions. Pharmaceutics.

[102] Emenike EC, Okorie CJ, Ojeyemi T, Egbemhenghe A, Iwuozor KO, Saliu OD (2023). From oceans to dinner plates: The impact of microplastics on human health. Heliyon.

[103] Hu M, Palić D (2020). Micro- and nano-plastics activation of oxidative and inflammatory adverse outcome pathways. Redox Biol.

[104] Xu Z, Huang L, Xu P, Lim L, Cheong KL, Wang Y (2024). Microplastic pollution in commercially important edible marine bivalves: A comprehensive review. Food Chem X.

[105] Daud A, Ishak H, Ibrahim E, Basir B, Birawida AB, Syam RC (2024). Environmental Health Risk of Microplastics Due to Consumption of Fish and Shellfish in the Coastal Area. Iran J Public Health.

[106] Walkinshaw C, Lindeque PK, Thompson R, Tolhurst T, Cole M (2020). Microplastics and seafood: lower trophic organisms at highest risk of contamination. Ecotoxicology and Environmental Safety.

[107] Eberhard T, Casillas G, Zarus GM, Barr DB (2024). Systematic review of microplastics and nanoplastics in indoor and outdoor air: identifying a framework and data needs for quantifying human inhalation exposures. Journal of Exposure Science & Environmental Epidemiology.

[108] Zhai X, Zheng H, Xu Y, Zhao R, Wang W, Guo H (2023). Characterization and quantification of microplastics in indoor environments. Heliyon.

[109] Wang B, Zhang N, Dai L, Zhang Y, Yin S, Yang X (2025). Nonylphenol promotes epithelial-mesenchymal transition in colorectal cancer cells by upregulating miR-151a-3p. Discov Oncol.

